# Exosomes containing miRNAs targeting HER2 synthesis and engineered to adhere to HER2 on tumor cells surface exhibit enhanced antitumor activity

**DOI:** 10.1186/s12951-020-00711-5

**Published:** 2020-10-27

**Authors:** Lei Wang, Xusha Zhou, Weixuan Zou, Yinglin Wu, Jing Zhao, Xiaoqing Chen, Grace Guoying Zhou

**Affiliations:** 1 School of Basic Medical Sciences, Guangzhou Medical University, Guangzhou, 511436 Guangdong China; 2 Shenzhen International Institute for Biomedical Research, Longhua District, 1301 Guanguang Rd. 3F Building 1-B, Silver Star Hi-tech Park, Shenzhen, 518116 Guangdong China

**Keywords:** Exosomes, HER2, miRNA, Targeted delivery, HER2-positive tumor, Intravenous injection

## Abstract

**Background:**

Exosomes are small, cellular membrane-derived vesicles with a diameter of 50–150 nm. Exosomes are considered ideal drug delivery systems with a wide range of applications in various diseases, including cancer. However, nonspecific delivery of therapeutic agents by exosomes in vivo remains challenging. Human epidermal growth factor receptor 2 (HER2) is an epidermal growth factor receptor tyrosine kinase, and its overexpression is usually associated with cell survival and tumor progression in various cancers. In this study, we aim to develop novel exosomes with dual HER2-targeting ability as a nanoparticle delivery vehicle to enhance antitumor efficacy in vivo.

**Results:**

Here, we report the generation of two kinds of exosomes carrying miRNAs designed to block HER2 synthesis, which consequently showed a distinct anti-tumor effect. The 293-miR-HER2 exosomes package and deliver miRNAs targeting HER2 to recipient cells to block HER2 synthesis. The anti-tumor effect of these exosomes on cancer cells dependent on HER2 for survival but do not affect cells that lack HER2 or that are engineered to express HER2 but are not dependent on it for survival. In contrast, 293-miR-XS-HER2 exosomes carry an additional peptide, which enables them to adhere to HER2 on the surface of cancer cells. Consequently, these exosomes preferentially enter these cells with surface expression of HER2 and further displayed a tumoricidal effect. The 293-miR-XS-HER2 exosomes are significantly more effective than the 293-miR-HER2 exosomes in shrinking HER2-positive tumors implanted in mice.

**Conclusions:**

Collectively, as novel antitumor drug delivery vehicles, HER2 dual-targeting exosomes exhibit increased target-specific delivery efficiency and can be further utilized to develop new nanoparticle-based targeted therapies. 
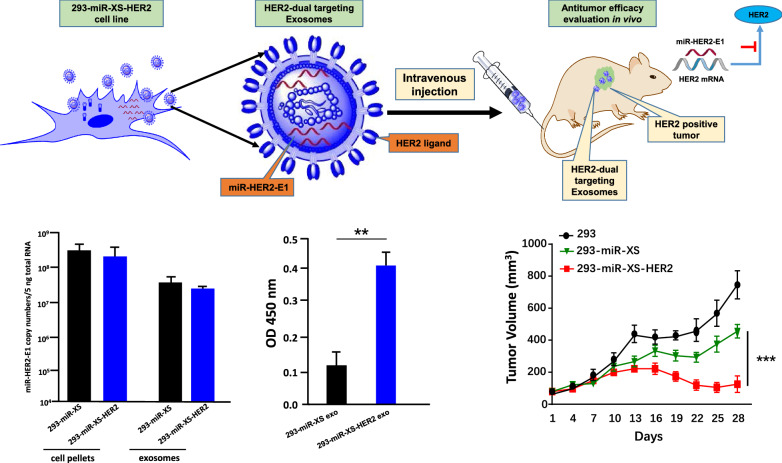

## Background

In this communication, we report the construction of exosomes designed to preferentially enter and ultimately displayed tumoricidal effects on cancer cells dependent on human epidermal growth factor receptor 2 (HER2) for their survival. The following concepts are relevant to this report:

HER2 is a member of the human epidermal growth factor receptor family [1–3]. The HER2 protein is expressed at high levels on the surface of human breast cancer cells. Its role in the oncogenic behaviors of these cells is supported by numerous observations [4–6]. Indeed, treatment of HER2-positive cells with antibodies was found to block G1-S cell cycle progression, decrease the protein levels of cyclin-dependent kinase 2 (CDK2), cyclin E, and CDK6 and reduce cyclin E-CDK2-associated kinase activity [7–10]. Administration of anti-HER2 antibodies is a standard of care treatment for human HER2-positive breast cancer patients [11–13].

Exosomes are small extracellular vesicles averaging 50–150 nm in diameter. They serve as a means of intercellular communication. Typically, they consist of structural proteins as well as selected proteins, miRNAs, mRNAs, and long noncoding RNAs [14–17]. The RNAs contain a short nucleotide sequence recognized by the proteins that transport them into the cytoplasm and package them into exosomes [18–20]. In an earlier study, this laboratory designed a miRNA targeting a major herpes simplex virus (HSV) regulatory protein. As predicted by the nucleotide packaging signal, the miRNAs were packaged in exosomes and, upon exposure to infected cells, significantly reduced virus yields [21].

This report consists of three parts. First, we report the construction of a miRNA targeting HER2 synthesis both in cells constitutively expressing HER2 and in cells transfected with a plasmid encoding HER2. Second, we report that the miRNA targeting the synthesis of HER2 reduced the viability of HER2-positive cancer cells both in cell culture and in implanted tumors. Last, we report that we enhanced the antitumor activity of these exosomes by binding to their surface a ligand with affinity for tumor cell surface HER2. These peptide-linked exosomes bind to the cell surface of and preferentially enter HER2-positive cells.

## Results

### Design and identification of miRNAs with EXO-motifs capable of suppressing HER2 synthesis

The objective of the first series of experiments was to design miRNAs targeting HER2. To this end, we constructed 7 miRNAs and selected the miRNA that most effectively in blocked HER2 synthesis in a HER2-positive cancer cell line and in a cell line transfected with a plasmid encoding HER2. The miRNA sequences were cloned into a miRNA expression vector named pcDNA6.2-GW/EmGFP-miR-neg downstream of an open reading frame encoding EGFP, as described in “Methods”.

In the initial screening, SK-OV-3, a cell line with high HER2 expression, and HEp-2, a HER2-negative cell line transfected with a plasmid encoding HER2, were transfected with the plasmids encoding the miRNAs. Of the 7 miRNAs tested (sequence shown in Additional file 1: Table S1), miRNAs No. 1 and No. 4 most effectively suppressed the accumulation of HER2 on protein level (Additional file 1: Fig. S1a, b). On the basis of these results, miRNAs No. 1 and No. 4 were selected for further studies and designated as miR-HER2-1 and miR-HER2-4, respectively.

**Fig. 1:**
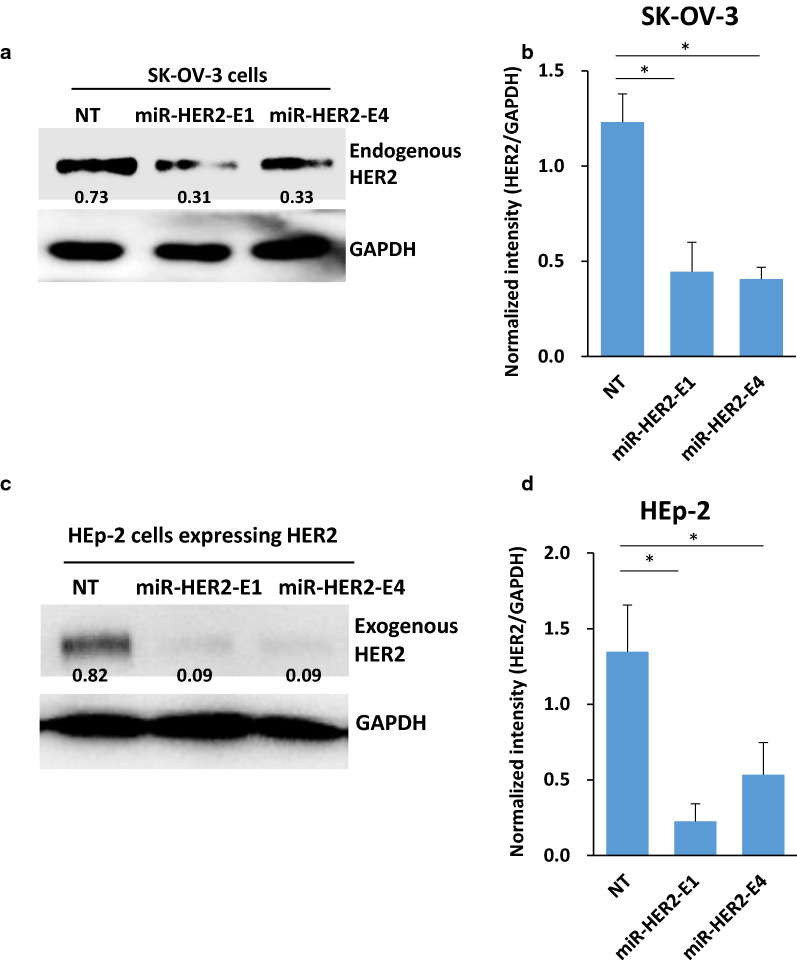
Downregulation of HER2 by miR-HER2-E1 and miR-HER2-E4 in SK-OV-3 and HEp-2 cells. **a** Immunoblotting analysis of HER2 protein levels of SK-OV-3 cells transfected with 0.5 μg of plasmids expressing miR-HER2-E1, miR-HER2-E4 or nontargeting (NT) miRNA. **b** The band densities of HER2 normalized to GAPDH indicating relative HER2/GAPDH expressing in SK-OV-3 cells were quantified and are presented as the mean ± standard deviation from three independent experiments. **c** HEp-2 cells were cotransfected with 0.5 μg of plasmid expressing miR-HER2-E1, miR-HER2-E4 or the NT miRNA and 0.2 μg of plasmid encoding His-tagged HER2. GAPDH served as a loading control. **d** The band densities of HER2 normalized to GAPDH indicating relative HER2/GAPDH expressing in HEp-2 cells were quantified and are presented as the mean ± standard deviation from three independent experiments. *p < 0.05

Next, we modified these two miRNAs by the addition of sequences containing exosome-packaging-associated motifs (EXO-motifs). The HER2 suppression efficacy of miR-HER2-E1 and miR-HER2-E4 was then retested in SK-OV-3 cells and in HEp-2 cells expressing HER2. The protein accumulation analyzed by immunoblotting showed that both miR-HER2-E1 and miR-HER2-E4 can significantly down-regulate the endogenous HER2 expression in SK-OV-3 cells (Fig. 1a, b) as well as exogenous HER2 expression in HEp-2 cells transfected with HER2 plasmid (Fig. 1c, d). On the basis of these results, we selected miR-HER2-E1 for further studies. The sequences of miR-HER2-1, miR-HER2-4, miR-HER2-E1 and miR-HER2-E4 are listed in "Methods".

### Production and characterization of exosomes-encapsulated miR-HER2-E1

**Fig. 2:**
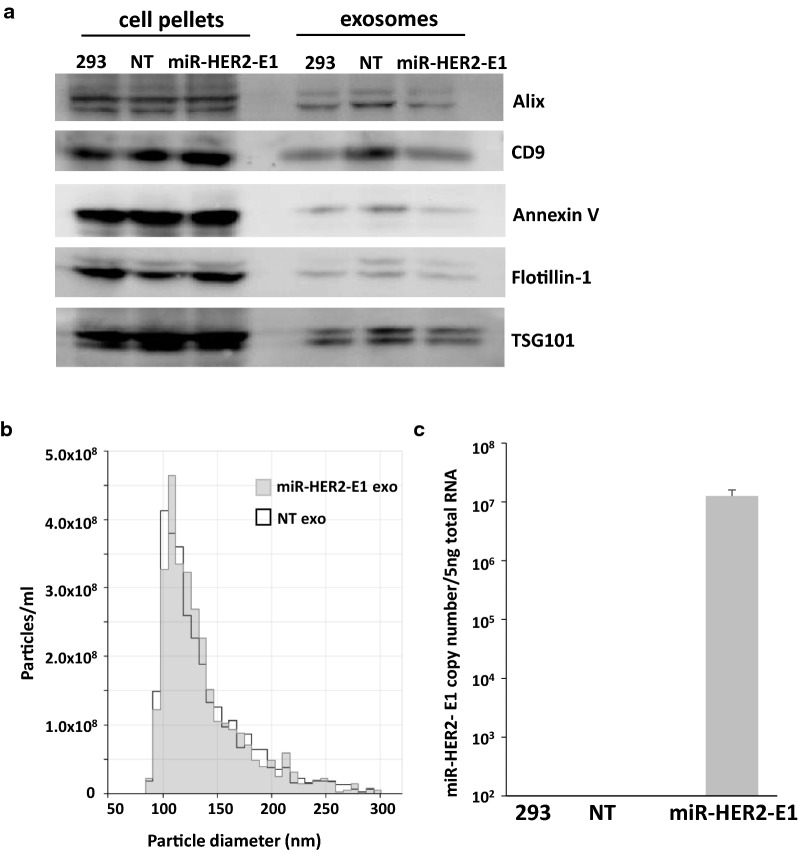
Characterization of exosomes containing miR-HER2-E1. **a** Immunoblotting analysis of exosomes and cells with antibodies against the exosome marker proteins Alix, CD9, Annexin V, Flotillin-1 and TSG101. **b** The particle size distribution and number of isolated exosomes extracted from HEK-293 cells transfected with miR-HER2-E1 or non-targeting (NT) plasmid were measured by Izon’s qNano technology (Izon). **c** Quantification of miR-HER2-E1 from purified exosomes by qPCR analysis. The amount of exosomal miR-HER2-E1 was quantified and normalized to the amount of 18S rRNA. The data reported are representative of three independent experiments. All quantitative data are presented as the mean ± standard deviation

In all experiments described in this report, we used exosomes produced by HEK-293 cells. The properties of these exosomes were investigated as described in brief below:

To assess the protein content of exosomes, purified exosomes derived from HEK-293 cells untreated (293) or transfected with non-targeting (NT) or miR-HER2-E1 plasmids were analyzed via Immunoblotting. Alix, CD9, Annexin V, Flotillin-1, and TSG101 were used as marker proteins of exosomes. As expected, the exosome-associated proteins were present in purified exosomes derived from untreated HEK-293, NT miRNA-transfected and miR-HER2-E1-transfected cells (Fig. 2a).

To determine the size distribution of exosomes, exosomes purified from HEK-293 cells transfected with miR-HER2-E1 were analyzed by Izon’s qNano technology as described in "Methods". The results (Fig. 2b) showed that exosomes from HEK-293 cells has similar size distributions as those cells transfected with NT or miR-HER2-E1, with diameters ranging from 75–150 nm.

Figure 2c shows the expression level of mature miR-HER2-E1 as measured by quantitative PCR (qPCR). As expected, high level of mature miR-HER2-E1 was detected in exosomes derived from HEK-293 cells transfected with miR-HER2-E1 plasmid, which indicated that Exo-motifs containing in miR-HER2-E1 contribute to package miRNA into exosomes. By contrast, the exosomes purified from parental HEK-293 cells, and the NT miRNA-transfected cells did not contain detectable amounts of miR-HER2-E1.

### Exosome-delivered miR-HER2-E1 decreases the accumulation of HER2 and reduces the viability of cells with high levels of HER2 expression

In this series of experiments, we examined whether miR-HER2-E1 produced in HEK-293 cells and delivered via exosomes effectively blocked the accumulation of HER2. We report 2 series of experiments below.

**Fig. 3:**
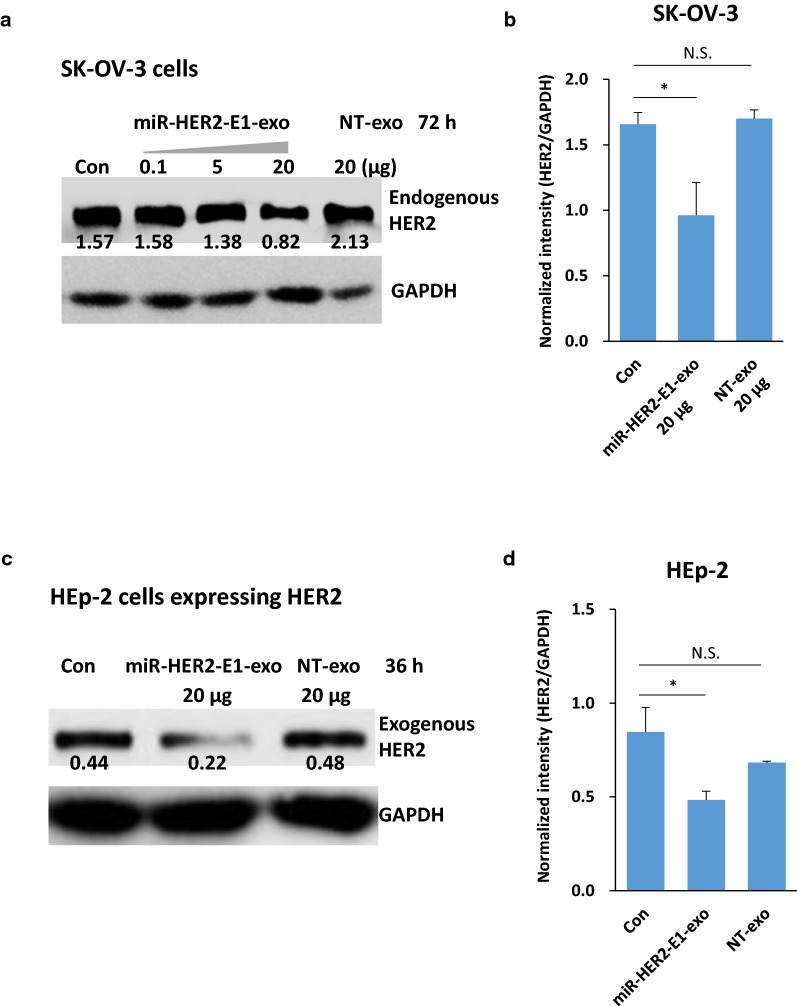
Inhibition of HER2 protein accumulation by exosome-delivered miR-HER2-E1. Immunoblotting analysis of HER2 protein levels in SK-OV-3 and HEp-2 cells treated with exosome-delivered miR-HER2-E1. **a** SK-OV-3 cells were left untreated (Con) or incubated with the indicated amounts of exosomes purified from HEK-293 cells transfected with miR-HER2-E1 or nontargeting (NT) miRNA. **b** The band intensity quantification shown at right represents the relative HER2/GAPDH expression levels in SK-OV-3 cell. **c** HEp-2 cells were transfected with the HER2 expression plasmid for 36 h and then either left untreated (Con) or incubated with 20 µg of purified exosomes produced by HEK-293 cells transfected with plasmids encoding the miR-HER2-E1 or the NT miRNAs. **d** The band intensity quantification shown at right represents the relative HER2/GAPDH expression levels in HEp-2 cells. The data reported are representative of three independent experiments. All quantitative data are presented as the mean ± standard deviation. *p < 0.05, N.S. indicates no significant difference

In the first series of experiments, the SK-OV-3 cells was exposed to different concentrations of purified exosomes carrying miR-HER2-E1 or purified exosomes produced by NT-transfected HEK-293 cells or remain no treatment (Con). It shows that the accumulation of HER2 decreased dose-dependently in SK-OV-3 cells exposed to exosomes containing miR-HER2-E1. Especially, HER2 expression in SK-OV-3 cells decreased significantly when treated with the highest concentration (20 μg) of purified exosomes-encapsulated miR-HER2-E1, while exosomes derived from NT transfected cells showed no effect on HER2 expression (Fig. 3a, b).

The second series of experiments was designed to determine whether miR-HER2-E1-expressing exosomes could also suppress the accumulation of exogenous HER2. HEp-2 cells were transfected with a plasmid encoding HER2 and were then exposed to purified exosomes mentioned as above. Figure 3c, d showed that the expression of exogenous HER2 was decreased in cells exposed to exosomes containing miR-HER2-E1 but not in exosomes derived from NT-transfected HEK-293 cells.

### Downregulation of HER2 expression via exosome-delivered miR-HER2-E1 can inhibit the viability of HER2-positive cancer cell by activation of apoptosis pathway

**Fig. 4:**
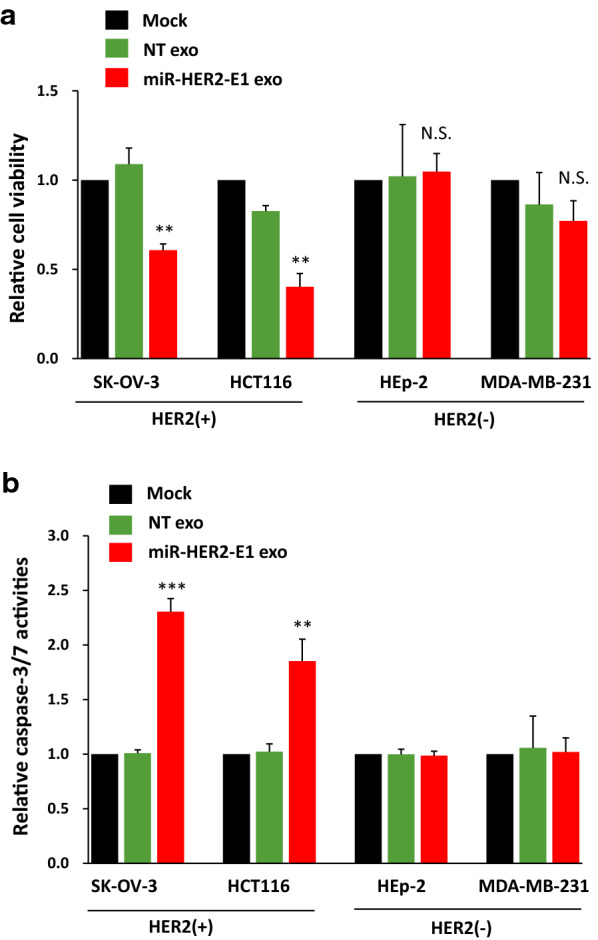
miR-HER2-E1 delivered to HER2-positive cancer cells via exosomes reduces cell viability by activation of caspase-3/7. **a** Effects of exosome-delivered miR-HER2-E1 on the viability of HER2-positive (+) cancer cells (SK-OV-3 and HCT116) and HER2-negative (−) cells (HEp-2 and MDA-MB-231) measured by the CCK8 assay. Results are expressed as the mean of the cell viability index ± standard deviation compared to the mock-treated control (as 100%). **b** Similarly, the activities of caspase-3/7 were assessed by a Caspase‐Glo 3/7 assay kit after 24 h-treatment of exosomes. The caspase-3/7 activity is expressed as fold change compared to the mock treated (Mock) group. Data are presented as mean ± standard error of three independent experiments. Statistically significant differences between miR-HER2-E1 exosomes and the mock-treated are indicated by an asterisk (**p < 0.01; ***p < 0.001) in the HER2-positive (+) group. N.S. indicates no significant difference

To test the hypothesis that exosome-delivered miR-HER2-E1 has tumoricidal effects on HER2-dependent cells by blocking replenishment of the protein in HER2-positive SK-OV-3 and HCT116 cancer cells as well as in HER2-negative MDA-MB-231 and HEp-2 cancer cells (Additional file 1: Fig. S2), the four cell lines were exposed to 1 µg of exosomes purified from HEK-293 cells transfected with miR-HER2-E1 respectively. And the relative cell viability was determined by a CCK8 assay. The results (Fig. 4a) showed the following:

i. SK-OV-3 cells exposed to exosomes carrying miR-HER2-E1 exhibited a viability of 60% relative to that of SK-OV-3 cells treated with exosomes carrying NT miRNA;
ii. HCT116 cells exposed to exosomes carrying miR-HER2-E1 exhibited a viability of 40% compared to the 90% viability of HCT116 cells treated with exosomes carrying NT miRNA;
iii. HEp-2 and MDA-MB-231 cells, the two types of HER2-negative cells, exhibited a viability of 90–100% upon exposure to exosomes carrying miR-HER2-E1.

These results suggest the following: (i) miR-HER2-E1 delivered by exosomes has anti-tumor effects on HER2-dependent cells by blocking the replenishment of the protein, and (ii) miR-HER2-E1 does not affect the viability of cells that are not dependent on HER2 for survival.

HER2 acts as an oncogene in solid tumor, its overexpression results in inhibition of caspase activation to prevent cell apoptosis [22]. To further investigate whether miR-HER2-E1 delivered by exosomes inhibits HER2-positive cell viability by restored the activity of executioner caspase-3/7, important markers of apoptosis, the four tumor cell lines were treated with purified exosomes produced by HEK-293 cells transfected with plasmids encoding miR-HER2-E1 or NT miRNA or remain mock treatment for 24 h. The activity of caspase-3/7 increased significantly in miR-HER2-E1 exosome treatment HER2-positive tumor cell lines compared with the mock treatment (Mock) assessed by a Caspase® Glo 3/7 assay kit (Fig. 4b), suggesting that the cell undergoes apoptosis. While there was no significant difference when treated with exosomes carried miR-HER2-E1 or mock in HER2-negative tumor cell lines. These results implicate that miR-HER2-E1 delivered by exosomes induces apoptotic cell death by downregulation of HER2 expression.

### Antitumor efficacy of miR-HER2-E1 delivered by administration of exosomes in vivo

**Fig. 5:**
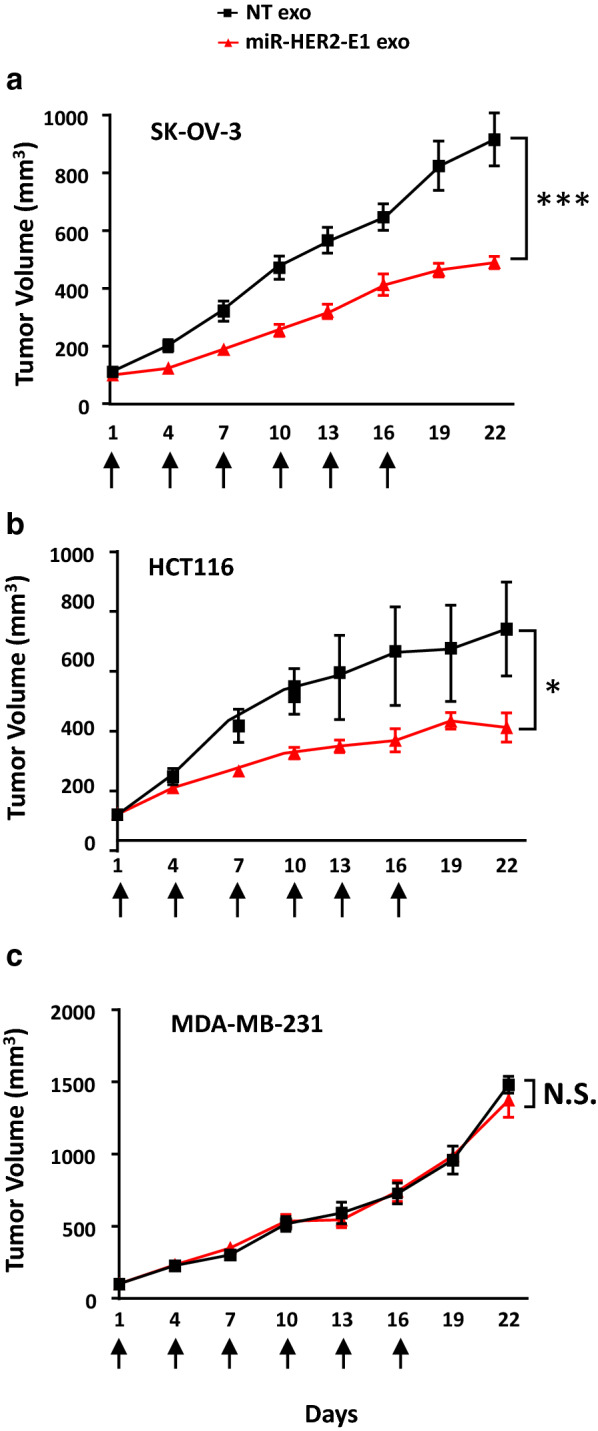
Antitumor efficacy of exosome-delivered miR-HER2-E1 in vivo. Nude mice bearing SK-OV-3 (**a**), HCT116 (**b**) or MDA-MB-231 (**c**) tumors (the average tumor size was 90 mm^3^ for each group) were injected intratumorally every three days, 6 times in total (indicated by arrow), with 10 μg of purified exosomes per injection. Tumor size was measured every three days. Results are shown as the mean tumor volume (mm^3^) ± standard deviation (n = 6). * and *** represent p < 0.05 and p < 0.001 compared with the NT exo group. N.S. indicates no significant difference

The SK-OV-3 (HER2-positive), HCT116 (HER2-positive) or MDA-MB-231 (HER2-negative) cells were used as tumor model for the in vivo study to evaluate the antitumor efficacy of miR-HER2-E1 delivered by exosomes via intratumoral administration. Ten microgram of exosomes purified from plasmid transfected HEK-293 cells were injected intratumorally every three days for a total 6 injections. The size of the tumors was measured every three days. The results (Fig. 5) showed that exosomes carrying miR-HER2-E1 caused a reduction in the volume of SK-OV-3 tumors (Fig. 5a) and HCT116 tumors (Fig. 5b) but not tumors induced by HER2-negative MDA-MB-231 tumor cells (Fig. 5c). These results indicated that miR-HER2-E1 delivered by exosomes can interfere HER2 expression (Additional file 1: Fig. S3) and specifically inhibit HER2-positive tumor growth in vivo, which were consistent with the results obtained in cell culture studies.

### Generation of stable cell line producing HER2 dual-targeting exosomes

The results of the experiments described above in the text showed that exosomes carrying miR-HER2-E1 entered both HER2-positive and HER2-negative cells but ultimately displayed tumoricidal effects only on HER2-positive cells. The objective of the studies described below was to enhance the entry efficiency of exosomes into HER2-positive cells. To achieve this objective, we generated two cell lines designed to produce novel exosomes. The surface of 293-miR-XS-HER2 exosomes carried a peptide that enabled these exosomes to adhere to HER2 on the surface of HER2-positive cells. In contrast, the surface of 293-miR-XS exosomes lacked the HER2 adhesion peptide. The miR-HER2-E1 was packaged into both types of exosomes. Details of the construction and characterization of the two generated cell lines are described in the “Methods”.

**Fig. 6.**
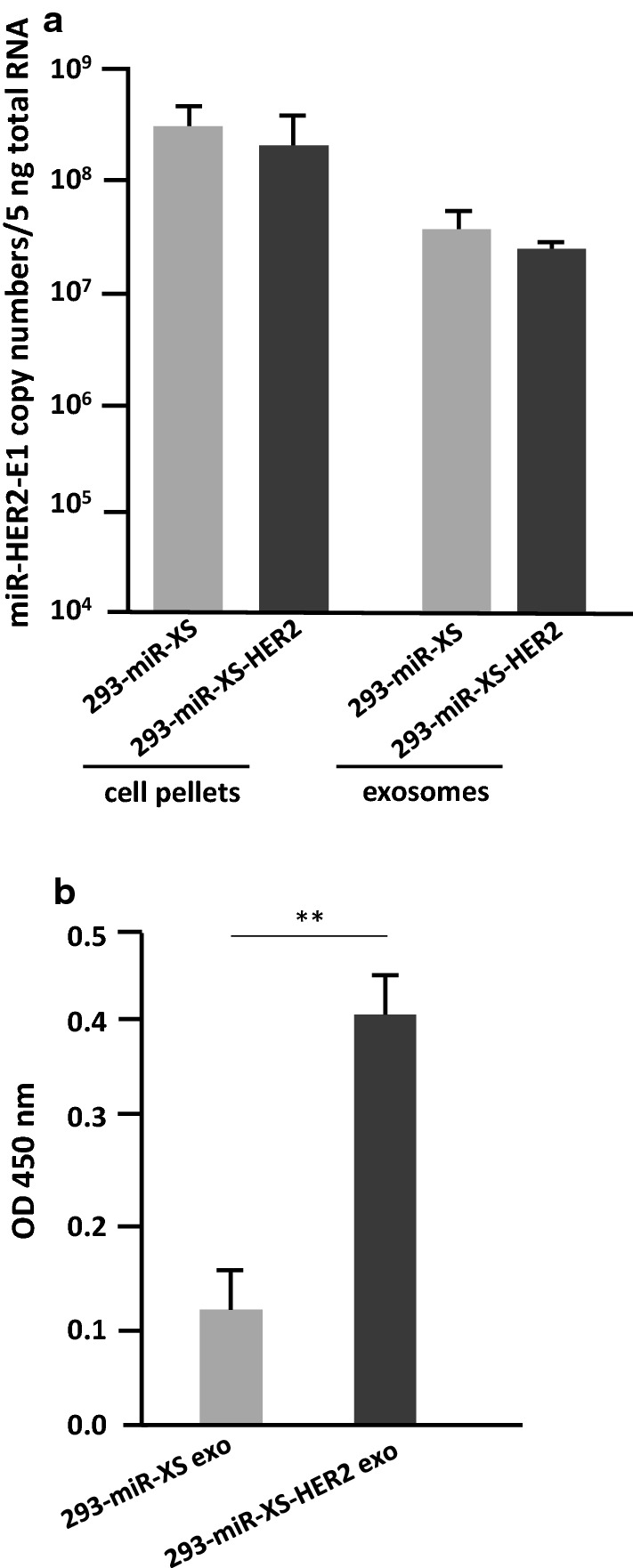
293-miR-XS-HER2 expressing both a ligand for tumor cell surface HER2 and a miRNA targeting HER2. **a** Accumulation of miR-HER2-E1 in stable cell lines. miR-HER2-E1 isolated from 293-miR-XS and 293-miR-XS-HER2 cell pellets and exosomes was quantified with normalization to the level of 18S rRNA. **b** The relative HER2 binding affinity of 293-miR-XS and 293-miR-XS-HER2 exosomes**.** The absorbance readings (OD 450 nm) are shown on the Y axis. Each result is presented as the mean ± standard deviation of three replicates. **p < 0.01

Firstly, we verified the presence of miR-HER2-E1 in exosomes produced by both cell lines. As shown in Fig. 6a, the exosomes produced by both cell lines contained mature miR-HER2-E1, as determined by analysis of cell pellets and purified exosomes.

Next, the exosomes produced by the two cell lines were purified and tested by ELISA to verify their ability to adhere to HER2 protein. As shown in Fig. 6b, purified exosomes produced by 293-miR-XS-HER2 cells preferentially bound HER2 protein. The results indicate that the dual-targeting 293-miR-XS-HER2 exosomes package miR-HER2-E1 targeting *HER2* gene and display a HER2-directed peptide on their surface, which enable 293-miR-XS-HER2 exosomes preferentially deliver HER2 miRNA into HER2 positive cells.

### Antitumor efficacy of exosomes carrying miR-HER2-E1 and adhering to HER2

**Fig. 7:**
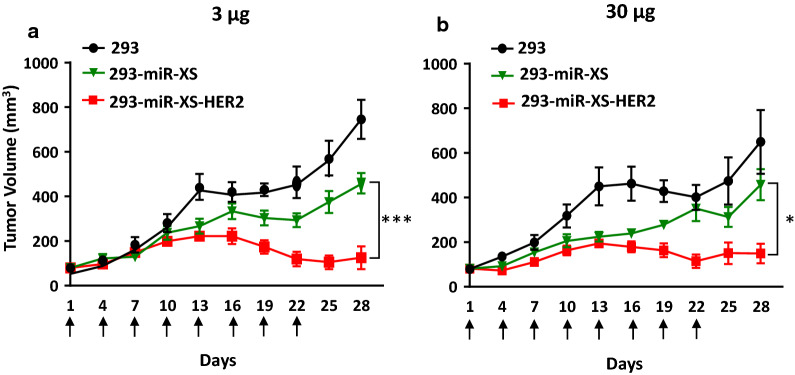
Antitumor efficacy of exosomes adhering to HER2 and expressing miR-HER2-E1. BALB/c-derived nude mice implanted with SK-OV-3 tumors with an average volume of 80 mm^3^ were injected intravenously with exosomes purified from the parental HEK-293 cell line (293), the miR-HER2-E1-expressing stable cell line (293-miR-XS) or the stable cell line with coexpression of the HER2 protein ligand and miR-HER2-E1 (293-miR-XS-HER2). Exosomes were injected 3 μg/animal (**a**) or 30 μg/animal (**b**) every three days, for a total of 8 injections (indicated by arrow). The tumor size was measured every three days. The results are shown as mean tumor volume (mm^3^) ± standard deviation (n = 6). * and *** represent p < 0.05 and p < 0.001 compared with the 293-miR-XS group

To verify whether the HER2-dual targeting exosomes (293-miR-XS-HER2) have improved antitumor efficacy compared with HER2 single targeting miRNA only (293-miR-XS) and non-targeting exosomes (293) in vivo by intravenous administration, HER2-positive tumor cells SK-OV-3 were transplanted into BALB/c nude mice. The results showed that compared to exosomes purified from HEK-293 or 293-miR-XS cells, exosomes purified from 293-miR-XS-HER2 cells were significantly more effective in reducing the growth of HER2-positive tumors (Fig. 7a, b). Moreover, the reductions in the sizes of tumors injected with 3 μg and 30 μg of 293-miR-XS-HER2 exosomes/mouse were virtually identical, suggesting that the 3 μg dose was close to or greater than the dose required to show tumoricidal effects on susceptible cells.

## Discussion

The studies presented in this report are the culmination of two discoveries made years ago. The first discovery was that cells synthesize small RNAs appropriately designated microRNAs (miRNAs) whose function is to bind to and terminate the translation of specific mRNAs. Control of specific functions by miRNAs is widespread, as illustrated by the observation that viruses also encode miRNAs. For example, HSV encodes diverse miRNAs, including miRNAs produced late in infection that regulate viral yields [23, 24].

The second major discovery centered on intercellular communication. It has long been known that neurons communicate via small vesicles [25, 26]. More recently, numerous studies have shown that cells communicate via small extracellular vesicles or exosomes carrying mRNAs, miRNAs, long noncoding RNAs and proteins [14–17]. These vesicles are secreted into the extracellular milieu by donor cells and are taken up by recipient cells. The developments that led to the present studies are twofold. First, the design and production of miRNAs targeting specific mRNAs has become commonplace. Second and equally important, numerous studies have shown that the packaging of RNAs into exosomes is not random but is based on short nucleotide sequences embedded in the RNAs [18–20]. Consequently, current technology enables the selective packaging of miRNAs designed to target a specific mRNA. For example, this laboratory has shown that a miRNA designed to target the mRNA encoding a major HSV regulatory protein can be efficiently packaged into exosomes and that its delivery to infected cells significantly impacted viral replication [21].

This report significantly extends the findings of earlier studies. The target in this study was the synthesis of HER2, a protein that defines the oncogenicity of a subtype of human breast cancer. In cancer cells, HER2 is located on the cell surface. Its role is implied by the observation that anti-HER2 antibodies selectively have tumoricidal effects on HER2-positive cancer cells [27]. Our studies showed that a miRNA designed to target HER2 mRNA and delivered to HER2-positive cells via exosomes blocked the replenishment of HER2 and ultimately showed anti-tumor effects on these cells by activation of apoptosis pathway. The data generated in this study also showed that this miRNA did not affect cells that did not express HER2 or that were induced to express HER2 by transfection of HER2 mRNA.

Moreover, this study went one step further. Tumors are heterogeneous and are likely to contain both HER2-positive cancer cells as well as HER2-negative cells. Although our studies indicated that exosomes carrying miRNAs targeting HER2 did not affect HER2-negative cells, it was nevertheless desirable to increase the uptake of exosomes carrying HER2-targeting miRNAs by HER2-positive tumor cells. To this end, we modified the exosomes with a surface peptide by which they bound to HER2 on the surface of cancer cells. Consequently, we significantly enhanced the uptake of exosomes carrying miRNAs directed against HER2 by HER2-positive cells.

## Conclusions

Taken together, our results provide proof of concept that dual-targeting exosomes, 293-miR-XS-HER2 exosomes, can be generated and exhibit increased antitumor activity in vivo by systemic delivery. The 293-miR-XS-HER2 exosomes achieve dual targeting of HER2 via a two-step approach. First, a HER2 binding peptide is displayed on the exosomal membrane, which enables specific entry into HER2-positive cancer cells. In the second step, a designed miRNA specifically targeting HER2 is released to downregulate HER2 protein expression and then further activate caspase-3/7 to promote apoptotic cell death. These data clearly indicate that as a novel antitumor drug delivery vehicle, HER2 dual-targeting exosomes exhibit increased efficiency of target-specific delivery and can be further utilized to develop new nanoparticle-based targeted therapies.

## Methods

### Purchased cell lines

The HEp-2 cell line (human laryngeal carcinoma cells) was purchased from the American Type Culture Collection (ATCC). The HEK-293 cell line (human embryonic kidney 293 cells) and SK-OV-3 cell line (human ovarian epithelial cancer cells) were purchased from the Cell Bank of the representative culture preservation committee of the Chinese Academy of Sciences (Shanghai, China). The HCT116 cell line (human colorectal carcinoma cells) and the MDA-MB-231 cell line (human breast cancer cells) were kindly provided by Professor Jun Du (Sun Yat-sen University, Guangzhou, China). HEp-2 cells were cultured in DMEM (high-glucose) supplemented with 5% (vol/vol) fetal bovine serum (FBS). HEK-293 and MDA-MB-231 cells were maintained in DMEM (high-glucose) containing 10% (vol/vol) FBS. SK-OV-3 cells were cultured in McCoy’s 5A medium supplemented with 10% (vol/vol) FBS. All culture media contained 100 U/ml penicillin and 100 μg/ml streptomycin. Cells were incubated in a humidified atmosphere containing 5% CO_2_ at 37 °C.

### Generation of stable cell lines

The stable cell line 293-miR-HER2, expressing miRNA targeting HER2, was generated by transfection of the miR-HER2-E1 plasmid into HEK-293 cells. Forty-eight hours after transfection, cells were selected by the addition of blasticidin (Solarbio Life Sciences) to a final concentration of 6 μg/ml. A cell colony with green fluorescent protein (GFP) expression was selected and cultured in complete medium with 6 μg/ml blasticidin. The cell line was monitored for the expression of GFP and miR-HER2-E1.

To generate a cell line producing exosomes that adhere to the surface of HER2-positive cells, 293-miR-HER2 cells were either infected with lentivector XSTP724PA-1 (XStamp HER2 ligand exosome HER2 receptor targeting lentivector) or infected with control lentivector XSTP710PA-1 according to the manufacturer's instructions (XStamp Technology, SBI: XSTP724PA-1/XSTP710PA-1). The two cell lines were renamed 293-miR-XS-HER2 and 293-miR-XS (control), respectively. The lentivector XSTP724PA-1 contains two copies of the HER2 ligand fused to the 5′ N-terminal signal sequence leader and fused in frame to the 3′ C-terminal C1C2 XStamp domain that directs the entire fusion protein to be displayed on the surface of secreted exosomes [28, 29]. The HER2 binding ability of the exosomes purified from 293-miR-XS-HER2 cells was confirmed by ELISA.

### Antibodies

The anti-HER2 (Cat. No. #2165S) antibody was purchased from Cell Signaling Technology. The anti-GAPDH, anti-Alix, anti-CD9, anti-Annexin V, anti-Flotillin-1, and anti-TSG101 antibodies have been described elsewhere [30].

### Plasmid construction

The miRNA sequences targeting the *HER2* gene were designed using BLOCK-iT™ RNAi Designer (Life Technologies) and synthesized by Ige Biotechnology (Guangzhou, China). The synthesized miRNA fragments were digested with the BamHI and XhoI restriction enzymes and cloned into the corresponding sites in the pcDNA6.2-GW/EmGFP-miR-neg control plasmid (Invitrogen). The miRNA sequences were as follows:

miR-HER2-1: 5′-AACTCAAGCAGGAAGGAAGGTGTTTTGGCCACTGACTGACACCTTCCTCTGCTTGAGTT-3'.

miR-HER2-4: 5′-TGTGAGAGCCAGCTGGTTGTTGTTTTGGCCACTGACTGACAACAACCATGGCTCTCACA-3'.

miR-HER2-E1: 5′-AACTCAAGCAGGAAGGAGGAGGTTTTGGCCACTGACTGAC CTCCTCCTCTGCTTGAGTT-3'.

miR-HER2-E4: 5′-TGTGAGAGCCAGCTGGAGGAGGTTTTGGCCACTGACTGACCTCCTCCATGGCTCTCACA-3'.

The sequences of the mature miRNAs are underlined.

miR-HER2-E1 and miR-HER2-E4 were modified versions of miR-HER2-1 and miR-HER2-4, respectively, as they contained EXO-motifs).

The HER2 expression plasmid containing the 3′-UTR sequence of the *HER2* gene was generated as follows. First, the His-tagged HER2 coding sequence was amplified by PCR using the following primers:

Forward, 5′-CCCAAGCTTATGGAGCTGGCGGCCTTGTG and.

Reverse, 5′-ATAAGAATGCGGCCGCTTATCAGTGATGGTGATGGTGATGCACTGGCACGTCCAGACCCAG.

The PCR fragment was then subcloned into pcDNA3.1(+) at the HindIII/NotI sites to generate pHER2-His. The 3′-UTR sequence was synthesized by Ige Biotechnology (Guangzhou, China), digested with the NotI and XbaI restriction enzymes and cloned into the corresponding sites in pHER2-His.

### Exosome isolation and quantification

HEK-293 cells (1 × 10^7^) seeded in a T150 flask were mock transfected or transfected with 10 μg of the NT or miR-HER2-E1 plasmid. After 4 h of incubation, the cells were rinsed extensively with phosphate-buffered saline (PBS) and incubated in serum-free medium for an additional 48 h. Cells of the stable cell lines were seeded in a T150 flask for 24 h, rinsed extensively with PBS and incubated in serum-free medium for another 48 h. Cell-free extracellular medium containing exosomes was harvested by centrifugation at 300 × g for 10 min to remove the cells. The supernatant was then centrifuged at 10,000 × g for 30 min to remove dead cells and cell debris. Finally, the clear supernatant was centrifuged for 70 min at 100,000×*g* to pellet the exosomes. All centrifugation steps were carried out at 4 °C. For immunoblotting, the pelleted exosomes were resuspended in RIPA buffer. For treatment of cells or mice, the pelleted exosomes were resuspended in PBS. The exosomes were quantified by the BCA method using an Enhanced BCA Protein Assay Kit (Beyotime) according to the manufacturer's instructions.

### Exosome size analysis

The size distribution of the exosomes was analyzed using the Izon qNano system (Izon, Christchurch, New Zealand; https://www.izon.com), which uses single-molecule electrophoresis to detect extracellular vesicles passing through a nanopore. The procedure yielded accurate particle-by-particle enumeration of exosomes ranging from 75 to 300 nm in diameter. Specifically, purified exosomes were diluted 1:10 in PBS containing 0.05% Tween 20, shaken vigorously, and measured by using an NP200 (A53942) nanopore aperture according to the manufacturer’s instructions. The results were analyzed using Izon Control Suite software v3.3 (Izon Science).

### Quantitative RT-PCR for miRNA

Total RNA from cells and suspended exosomes was isolated using TRIzol reagent (Thermo Fisher Scientific) and TRIzol LS reagent (Thermo Fisher Scientific) according to the manufacturer’s instructions. The miRNAs tested were reverse transcribed from 50 ng of total RNA in duplicate with specific stem-loop primers as described in the TaqMan miRNA reverse transcription kit instructions (Applied Biosystems, Inc.). miRNA expression was measured by real-time PCR using a TaqMan Universal Master Mix II kit purchased from Applied Biosystems, Inc. The miRNA copy number was normalized to that of cellular 18S rRNA. The primers specific for miR-HER2-E1 were designed according to Chen et al. [31] and synthesized by Ige Biotechnology. The sequences follow:

miR-HER2-E1 stem-loop primer,

5′-GTCGGTCGTATCCAGTGCAGGGTCCGAGGTATTCGCACTGGATACGACCTCCTCCT-3′;

Forward primer, 5′-AACCAAGCAGGAAGGAGG-3′;

Reverse primer, 5′-GTGCAGGGTCCGAGGT-3′;

Probe, 5′-(6-FAM) TCGCACTGGATACG (MGB)-3’.

### Plasmid transfection

SK-OV-3 or HEp-2 cells were seeded in 12-well plates at 2.5 × 10^5^ cells per well. Cells were transfected with 0.5 μg of plasmids expressing miR-HER2-E1, miR-HER2-E4 or non-targeting (NT) miRNA or for HEp-2 cells were contransfected with 0.2 μg of a plasmid encoding His-tagged HER2 using Lipofectamine 2000 reagent (Thermo Fisher Scientific) following manufacturer's instructions. Cells were harvested at 72 h after transfection and used for immunoblotting analysis.

### Exosomes incubation

SK-OV-3 cells at 2.5 × 10^5^ cells per well were exposed to different concentrations of purified exosomes (0.1 μg, 5 μg, or 20 μg) carrying miR-HER2-E1 or purified exosomes (20 μg) produced by NT-transfected HEK-293 cells or remain no treatment (Con) for 72 h. For HEp-2 cells at a density of 2.5 × 10^5^ per well were transfected with 0.2 μg of a plasmid encoding His-tagged HER2 for 36 h and then incubated 20 µg of purified exosomes produced by HEK-293 cells transfected with plasmids encoding the miR-HER2-E1 or the NT miRNAs or remain no treatment for another 36 h. The cells were then harvested for endogenous and exogenous HER2 expression by immunoblotting analysis.

### Immunoblotting assay

Cell pellets or purified exosomes were harvested and lysed with RIPA lysis buffer (Beyotime) supplemented with the protease inhibitor phenylmethylsulfonyl fluoride (PMSF) (1 mM; Beyotime) and a phosphatase inhibitor (Beyotime). Cell lysates and exosomes were heat denatured at 100℃ incubator for 10 min, separated by 10% or 8% SDS-PAGE and transferred to polyvinylidene difluoride membranes (Millipore). The proteins were identified by incubation with the appropriate primary antibody and then with an HRP-conjugated secondary antibody (Pierce). Immunoreactions were visualized with ECL reagent (Pierce), and images were acquired using a ChemiDoc Touch Imaging System (Bio-Rad) and analyzed with Image Lab software. The densities of the corresponding bands were quantified using ImageJ software.

### Cell viability assay

SK-OV-3, HCT116, HEp-2 and MDA-MB-231 cells were seeded into 96-well plates at a density of 1 × 10^4^ cells per well one day before exposure to exosomes. Cells in triplicate wells were mock treated (Mock) or incubated with 1 µg of purified exosomes produced by HEK-293 cells transfected with plasmids encoding either miR-HER2-E1 or the nontargeting (NT) miRNA. After 72 h of incubation, the relative cell viability was determined by a CCK8 assay according to the manufacturer’s protocol.

### Caspase-3/7 activity detection

SK-OV-3, HCT116, HEp-2 and MDA-MB-231 cells were seeded at a density of 2 × 10^3^ cells per well on 96-well plates, and allowed to adhere overnight. Cells in triplicate wells were mock treated (Mock) or incubated with 1 µg of purified exosomes produced by HEK-293 cells transfected with plasmids encoding either miR-HER2-E1 or the nontargeting (NT) miRNA, and the plates were incubated at 37 °C with 5% CO_2_ for 24 h. After treatment, the Caspase-Glo® 3/7 (Promega) was prepared according to manufacturer’s guidelines, and 100 μL of the reagent was added per well and incubated for 1 h at room temperature in the dark. The luminescent signal was recorded by a multimode microplate reader (Synergy Multi-Mode Reader, BioTek).

### Animal models

BALB/c nude mice at 6–7 weeks of age were purchased from Vital River Laboratory Animal Technologies Co., Ltd. (Beijing, China). The nude mice were injected subcutaneously in the flanks with 5 × 10^6^ SK-OV-3, HCT116 or MDA-MB-231 cells respectively. In the exosome-delivered miR-HER2-E1 treatment, mice with tumors having an average volume of 90 mm^3^ were injected intratumorally with 50 μl containing 10 μg of purified exosomes per injection. Each tumor-bearing animal was injected on days 1, 4, 7, 10, 13, and 16, for a total of 6 injections. The size of tumors was measured on days 1, 4, 7, 10, 13, 16, 19 and 22. In the exosome-delivered 293-miR-XS-HER2 treatment nude mice derived from BALB/c were injected subcutaneously into flanks with 5 × 10^6^ SK-OV-3 cells. Tumors averaging 80 mm^3^ were injected intravenously on days 1, 4, 7, 10, 13, 16, 19 and 22 with 3 μg/animal or 30 μg/animal of exosomes purified from 293-miR-XS-HER2, 293-miR-XS or parental HEK-293 cells. The sizes of tumors were measured on days 1, 4, 7, 10, 13, 16, 19, 22, 25 and 28 using a caliper, and the volume was calculated as (length × width^2^) × 0.5.

### ELISA

Exosomes purified from the 293-miR-XS or 293-miR-XS-HER2 stable cell lines were coated (1 μg/well) in triplicate wells of 96-well ELISA plates (Corning). After the coating solution was removed, nonspecific binding sites were blocked by incubation with 2% BSA at 37 °C* for *1 h. The plates were rinsed, exposed to HER2 protein (Sino Biological, China) for 2 h, rinsed again, and reacted with the HRP-conjugated rabbit anti-HER2 antibody (Cat. No. 1004-R205, Sino Biological) for an additional 1 h. The plates were then rinsed again and exposed to TMB for color development. The reaction was terminated by the addition of stop solution. The plates were read in a BioTek microplate reader at a wavelength of 450 nm.

## Supplementary information

**Additional file 1: Figure S1.** Screening knock-down efficacy of transfected plasmids expression miR-HER2. **Figure S2.** The endogenous expression of HER2 in different tumor cell lines. **Figure S3.** Downregulation of HER2 expression with miR-HER2-E1 in SK-OV-3 xenograft model.

## Data Availability

Data sharing is not applicable to this article, as no datasets were generated or analyzed during the current study.

## Abbreviations

HER2: Human epidermal growth factor receptor 2
miRNAs: MicroRNAs
HSV: Herpes simplex virus
CDK2: Cyclin-dependent kinase 2
EXO-motifs: Exosome-packaging-associated motifs
NT: Nontargeting
i.t: Injected intratumorally
i.v: Injected intravenously
FBS: Fetal bovine serum
PMSF: Phenylmethylsulfonyl fluoride
BSA: Bovine serum albumin
HRP: Horseradish peroxidase
TMB: Tetramethyl benzidine substrate
