# Supplementary material for: Exosomes containing miRNAs targeting HER2 synthesis and engineered to adhere to HER2 on tumor cells surface exhibit enhanced antitumor activity
Source: J Nanobiotechnology. 2020 Oct 27;18:153. doi: 10.1186/s12951-020-00711-5 (PMC7592554; doi:10.1186/s12951-020-00711-5)
Supplement: Supplementary file 1 — Additional file 1: Figure S1. Screening knock-down efficacy of transfected plasmids expression miR-HER2. Figure S2. The endogenous expression of HER2 in different tumor cell lines. Figure S3. Downregulation of HER2 expression with miR-HER2-E1 in SK-OV-3 xenograft model. [file 12951_2020_711_MOESM1_ESM.pdf]

Additional file 1

Table S1. The designed sequences of miRNAs targeting HER2

No. of miRNAs	Mature miRNA sequences
**miR-HER2-1**	5'-AACTCAAGCAGGAAGGAAGGT-3'
**miR-HER2-2**	5' AGTACTAGGTTTCAGGGACAG-3'
**miR-HER2-3**	5'-AAAGCCTGGATACTGACACCA-3'
**miR-HER2-4**	5'-TGTGAGAGCCAGCTGGTTGTT-3'
**miR-HER2-5**	5'- AAAGCTCTCCGGCAGAAATGC-3'
**miR-HER2-6**	5'-TGTATAGGTAACCTGTGATCT-3'
**miR-HER2-7**	5'-TGTACTTCCGGATCTTCTGCT-3'

Figure S1

a SK-OV-3 (HER2-high expressing cell line)

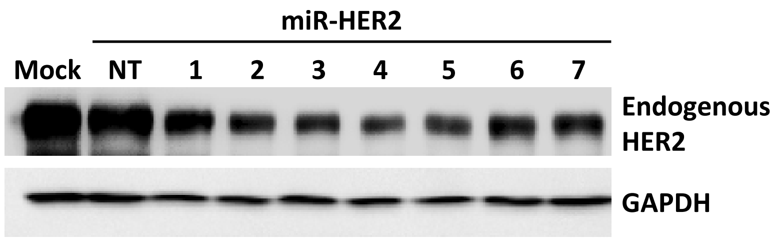

b HEp-2（HER2-low expressing cell line ）

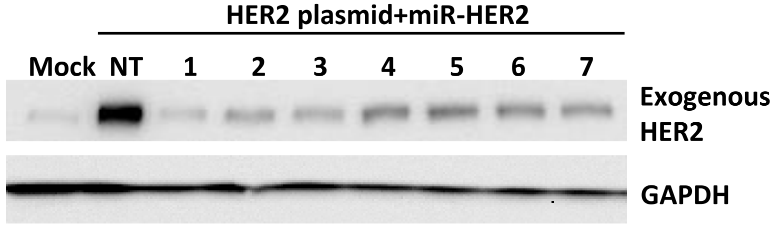

Figure S1. **a.** Immunoblotting analysis of HER2 protein levels of SK-OV-3 cells transfected with plasmids expressing miR-HER2 1-7 or nontargeting (NT) miRNA.
b. HEp-2 cells were cotransfected with plasmid expressing miRNA targeting HER2 (1-7) or the NT miRNA and plasmid encoding HER2 with His tag.

Figure S2

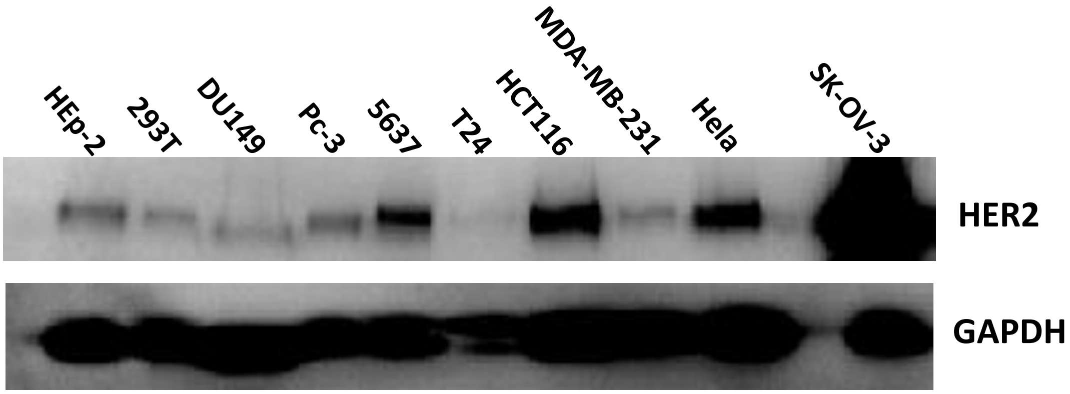

Figure S2. Endogenous expression of HER2 in different tumor cell lines. Immunoblotting analysis was performed using anti-HER2 antibodies. GAPDH served as a loading control.

Figure S3

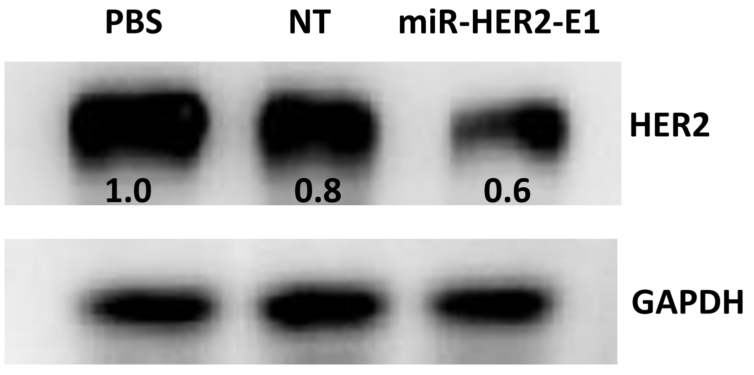

Figure S3. miR-HER2-E1 downregulate HER2 expression in Figure 5a SK-OV-3 xenograft. SK-OV-3 tumor xenografts established as shown in Figure 5a were removed from mice administered with PBS, NT exo and miR-HER2-E1 exo at the end of experiments. The tumor homogenate was lysed by RIPA followed by evaluation of HER2 expression via Immunoblotting assay. The band densities of HER2 in NT exo group and miR-HER2-E1 are normalized to PBS group. GAPDH served as a loading control.
